# Does the coronal plane alignment of the ankle and subtalar joints normalize after total knee arthroplasty?

**DOI:** 10.1186/s43019-025-00272-7

**Published:** 2025-05-08

**Authors:** Katsuki Yamaguchi, Tatsuya Sakai, Masanori Fujii, Satoshi Takashima, Shuichi Eto, Yosuke Matsumura, Satomi Nagamine, Hirofumi Tanaka

**Affiliations:** 1 Department of Orthopaedic Surgery, Faculty of Medicine, Saga University, 5-1-1 Nabeshima, Saga, 849-8501 Japan; 2 Department of Orthopaedic Surgery, National Hospital Organization Saga National Hospital, 1-20-1 Hinode, Saga, 849-0923 Japan; 3 Department of Orthopaedic Surgery, Hyakutake Orthopaedics and Sports Clinic, 4-2-15 Mizugae, Saga, 840-0054 Japan

**Keywords:** Total knee arthroplasty, Hindfoot alignment, Ankle osteoarthritis, Subtalar joint, Coronal alignment

## Abstract

**Background:**

Total knee arthroplasty (TKA) alters the lower extremity alignment, potentially affecting adjacent joints such as the ankle and subtalar joints. However, the relationship between changes in hindfoot alignment and ankle osteoarthritis (OA) after TKA remains incompletely understood. The purpose of this study was to clarify whether ankle and subtalar alignment normalizes after TKA and to identify factors associated with persistent malalignment.

**Methods:**

We retrospectively analyzed 331 patients who underwent unilateral mechanical alignment (MA) TKA for knee osteoarthritis. A control group of 40 healthy subjects was used to define normal alignment ranges. Whole-leg anteroposterior weight-bearing radiographs were obtained preoperatively and 2 months postoperatively. Alignment parameters included the hip–knee–ankle angle (HKA), tibiotalar tilt angle (TTA), tibial plafond inclination angle (TPIA), talar inclination angle (TIA), and hindfoot alignment angle (HAA). Pre- and postoperative values were compared using the Wilcoxon signed-rank test, and changes in the proportion of patients within the normal range were determined. Wilcoxon rank-sum tests and chi-squared tests were used for group comparisons, and multivariate logistic regression identified independent predictors of persistent malalignment.

**Results:**

HKA improved after TKA (−12° to −2.0°), with corresponding improvements in TPIA (99° to 94°) and TIA (99° to 95°) (all *p* < 0.001), indicating a significant correction toward neutral alignment. The proportion of patients within normal range increased postoperatively from 16% to 85% for HKA, 26% to 67% for TPIA, 24% to 64% for TIA, and 65% to 73% for HAA. Multivariate analysis identified ankle OA (odds ratio [OR] = 6.62 for TTA), female sex (OR = 2.32 for TPIA; OR = 3.19 for TIA), and varus knee alignment (OR = 2.81 for TIA) as independent predictors of persistent malalignment.

**Conclusions:**

MA-TKA facilitates partial normalization of coronal hindfoot alignment, particularly at the tibial plafond and talus. However, female sex, varus knee deformity, and pre-existing ankle OA independently limit full correction. These findings highlight the biomechanical interdependence between the knee and hindfoot and may guide surgical decision-making and patient-specific alignment strategies.

## Background

Total knee arthroplasty (TKA) is a well-established treatment for end-stage knee osteoarthritis (OA), and its use continues to increase with an aging population [1]. Mechanical alignment (MA) TKA aims to restore neutral alignment by correcting varus or valgus deformities of the lower extremity. Several studies have shown that such realignment also affects hindfoot alignment [2, 3], potentially influencing the onset and progression of adjacent joint pain and OA, particularly in the ankle [4, 5]. In contrast, kinematically aligned (KA) TKA—designed to restore the native joint line and ligamentous balance—has gained popularity [6]. Therefore, understanding how different alignment strategies affect hindfoot alignment is of increasing clinical relevance. The mechanical axis of the lower extremity extends from the hip to the ankle and passes through multiple joints; therefore, deformities at the knee can alter distal alignment and joint loading [7, 8]. Hindfoot malalignment can subsequently affect ankle kinematics and contribute to degenerative changes [9]. However, the biomechanical consequences of these alignment changes remain incompletely understood.

Previous studies have shown that 24–35% of patients undergoing TKA have concomitant ankle OA, which may adversely affect outcomes, with a 22% incidence of postoperative onset or progression of ankle OA [4, 10]. Residual knee deformity, subtalar joint stiffness, and limited compensatory capacity may impair hindfoot realignment after TKA [2, 3, 5, 11, 12]. In varus knee OA, hindfoot valgus often develops as a compensatory mechanism mediated by subtalar joint mobility [13]; failure of this mechanism may lead to abnormal ankle loading, exacerbating pain and degenerative changes. Notably, 11–25% of patients remain dissatisfied after TKA [4, 5], and ankle pain may persist, worsen, or recur postoperatively [4, 11, 14], or even after high tibial osteotomy [15]. Chang et al. reported that valgus correction ≥ 10° may cause ankle malalignment and pain [16], whereas others have shown symptomatic improvement with correction of knee alignment [5, 17]. Despite these findings, the clinical relevance of radiographic changes in ankle alignment remains unclear, and no studies have clearly defined the underlying pathophysiology [4, 18]. A better understanding of these relationships is essential to anticipate adjacent joint pathology and optimize postoperative outcomes.

To our knowledge, no study has used healthy individuals as a reference group to evaluate hindfoot alignment after TKA [19]. By establishing normal reference values, it is possible to objectively assess whether achieving neutral limb alignment with TKA also restores neutral hindfoot alignment. This approach may also help identify risk factors for residual malalignment and inform targeted interventions.

In this study, we retrospectively assessed hindfoot alignment using defined normative values derived from healthy controls to determine: (1) the incidence and radiographic characteristics of ankle OA in patients undergoing TKA, (2) whether lower extremity realignment via MA-TKA leads to normalization of hindfoot alignment, and (3) factors associated with residual hindfoot malalignment after TKA. We hypothesized that MA-TKA would improve coronal ankle and subtalar alignment, but that normalization would be limited in patients with pre-existing ankle OA.

## Methods

### Patients

**Fig. 1:**
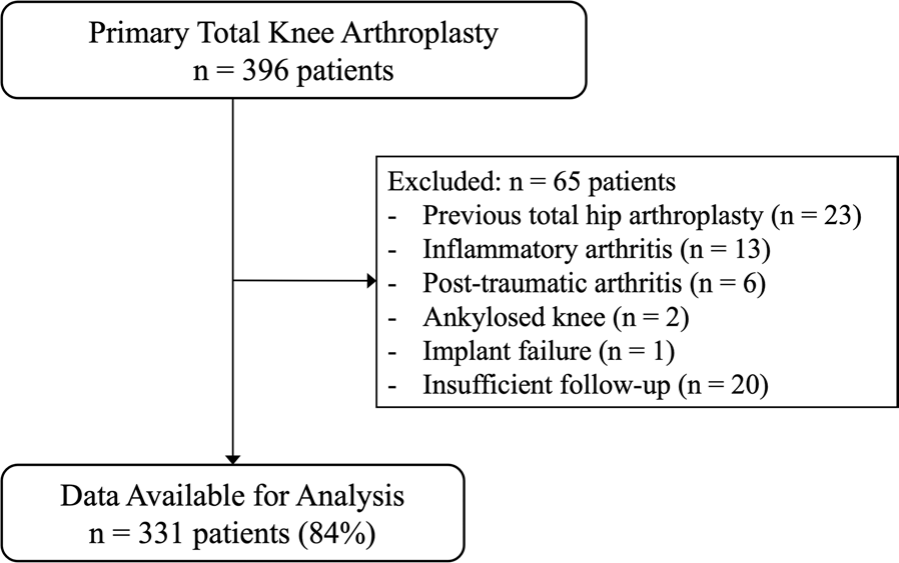
Flow diagram illustrating the patient selection process for this study

Clinical and radiographic data for all TKA procedures performed between June 2015 and January 2021 were retrospectively reviewed using a prospectively collected institutional database. All procedures were performed using an MA technique. Inclusion criteria for this study were: patients aged ≥ 18 years, diagnosed with primary knee OA, and undergoing initial unilateral TKA. A total of 396 patients (396 knees) met these criteria. To reliably evaluate changes in hindfoot alignment following lower extremity realignment, the study was limited to patients with primary OA without adjacent joint pathology. The exclusion criteria were previous total hip arthroplasty or other surgery on the affected limb (23 patients), secondary knee OA due to inflammatory arthritis (13 patients), post-traumatic arthritis (6 patients), ankylosed knee (2 patients), postoperative implant failure (1 patient), and loss to follow-up (20 patients). After applying these criteria, 331 patients (331 knees; 84%) were included in this study (Fig. 1). Demographic data, including age, sex, body mass index (BMI), and laterality of the affected knee, were obtained via medical chart review (Table 1). The cohort included 81 males and 250 females with a median age of 75 years and a median BMI of 26 kg/m^2^. Surgery was performed on the right knee in 168 patients and on the left knee in 163 patients. Preoperatively, 286 patients had a varus knee deformity and 45 patients had a valgus deformity.

**Table 1:** Demographic data of patient and control groups

Parameters	TKA patients (*n* = 331 patients)	Controls (*n* = 40 patients)
Age^*^ *(years)*	75 (36–91)	23 (22–28)
Sex^†^ (male:female)	81 (24): 250 (76)	20 (50): 20 (50)
Body mass index^*^ (kg/m^2^)	26 (15–40)	21 (17–26)
Side^†^ (right:left)	168 (51): 163 (50)	40 (50): 40 (50)
Knee deformity^†^ (varus:valgus)	286 (86): 45 (14)	49 (61): 31 (39)

^*^Median (range); ^†^number (%)

A control group of 40 healthy medical students (80 knees) with no lower extremity complaints who consented to radiographic assessment for a lower extremity alignment study was also recruited. Each participant underwent whole-leg standing radiographs of both lower extremities. The control group consisted of 20 males and 20 females with a median age of 23 years and a median BMI of 21 kg/m^2^ (Table 1).

This retrospective study was approved by the institutional review board (approval number: 2022–06-R-05).

### Radiographic measurements

**Fig. 2:**
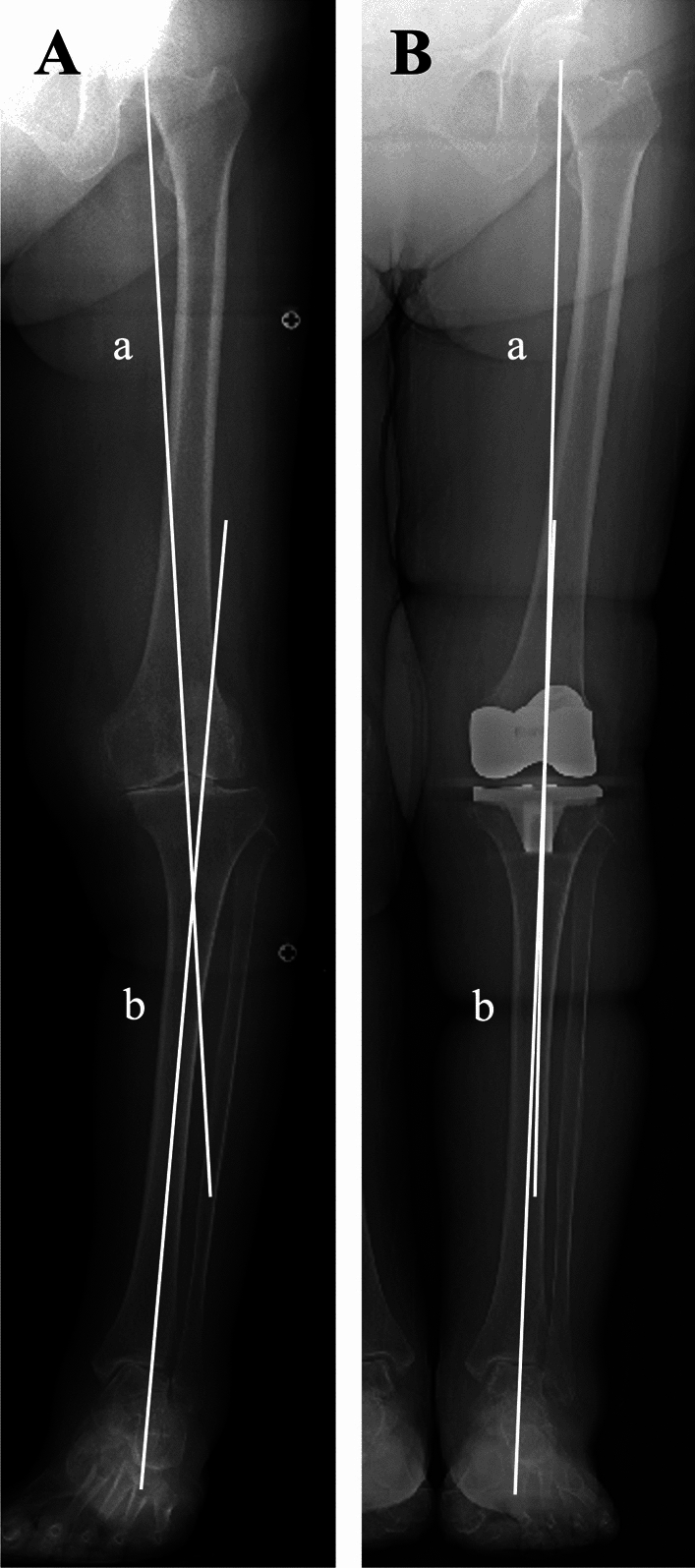
Whole-leg anteroposterior weight-bearing radiographs obtained (**A**) preoperatively and (**B**) 2 months after total knee arthroplasty using a mechanical alignment technique. Global alignment of the lower extremity was assessed using the hip–knee–ankle (HKA) angle, defined as the angle between the mechanical axis of the femur (line a) and that of the tibia (line b)

Whole-leg anteroposterior weight-bearing radiographs of the lower extremities were obtained preoperatively and 2 months postoperatively (Fig. 2). During the radiographic examination, the foot rotation was standardized using a foot positioning template on the platform. All radiographs were digitally acquired and analyzed using picture archiving and communication system (PACS) software.

**Fig. 3:**
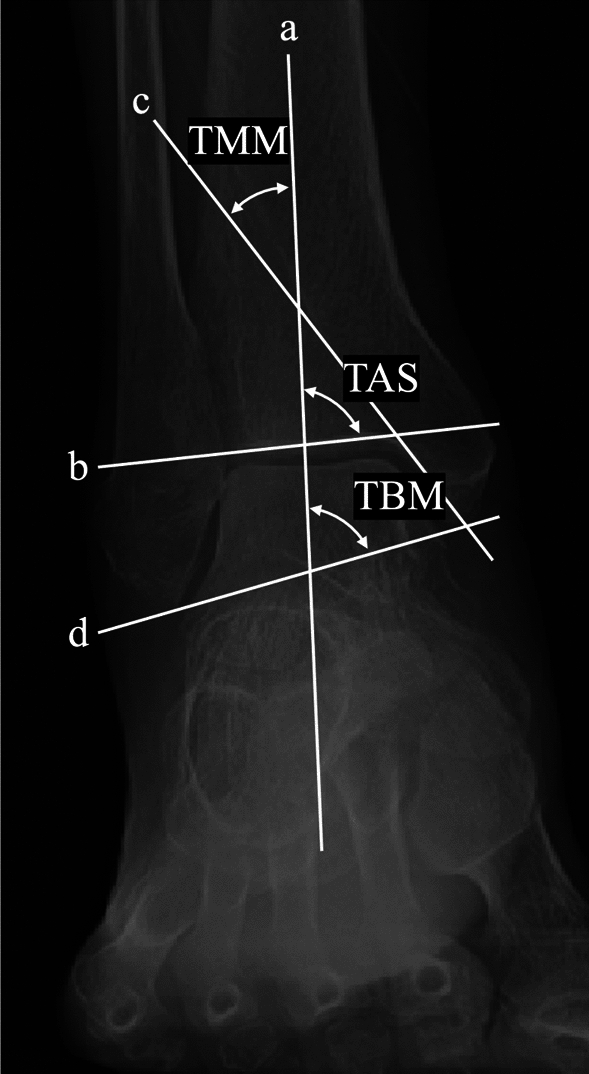
Radiographic measurements of morphometric parameters on whole-leg anteroposterior weight-bearing radiographs. Line (**a**) represents the mechanical axis of the tibia, (**b**) the subchondral plate of the distal tibial articular surface, (**c**) the subchondral plate of the medial malleolus articular surface, and (**d**) the line connecting the tips of both malleoli. The tibial anterior surface angle (TAS) is defined as the angle between lines (**a**) and (**b**), the tibial medial malleolus angle (TMM) is the angle between lines (**a**) and (**c**), and the tibial bimalleolus angle (TBM) is the angle between lines (**a**) and (**d**)

**Fig. 4:**
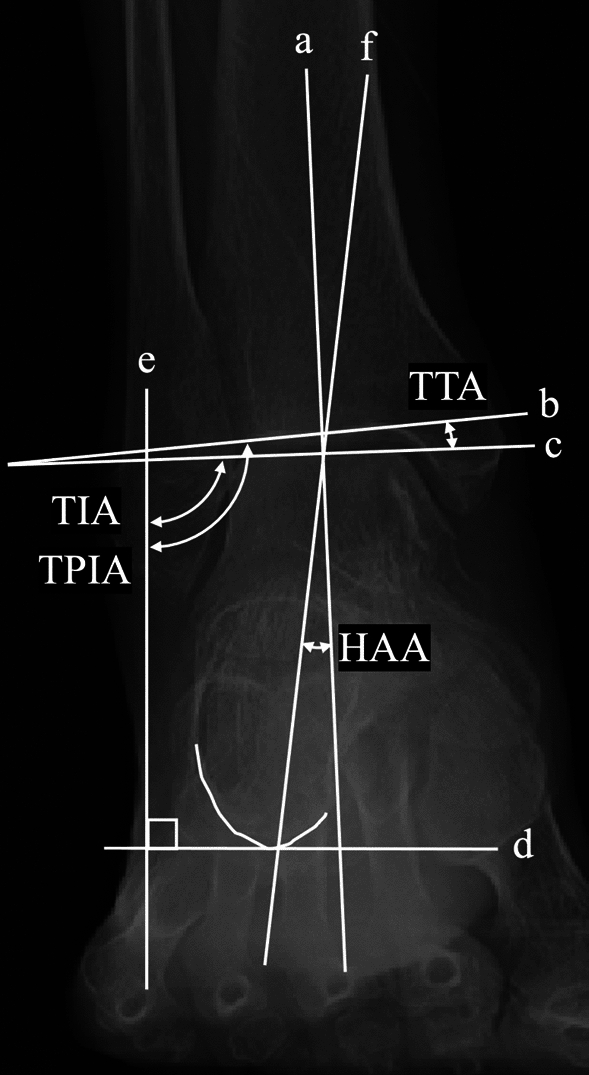
Radiographic measurements of ankle and subtalar joint alignment parameters on whole-leg anteroposterior weight-bearing radiographs. Line (**a**) represents the mechanical axis of the tibia, (**b**) the subchondral plate of the distal tibial articular surface, (**c**) a tangent to the talar dome, (**d**) a horizontal reference line parallel to the ground, (**e**) a line perpendicular to the ground, and (**f**) a line extending from the lowest point of the calcaneus to the center of the talar dome. The tibiotalar tilt angle (TTA) is defined as the angle between lines (**b**) and (**c**), the tibial plafond inclination angle (TPIA) is the angle between lines (**b**) and (**e**), the talar inclination angle (TIA) is the angle between lines (**c**) and (**e**), and the hindfoot alignment angle (HAA) is the angle between lines (**a**) and (**f**)

All measurements were performed in accordance with previously established protocols. Ankle OA was graded according to the Takakura–Tanaka classification, with stage ≥ 2 considered indicative of OA [9, 20]. Three bone morphometric parameters were measured (Fig. 3): tibial anterior surface angle (TAS), an indicator of ankle varus deformity; tibial medial malleolus angle (TMM), the distal opening angle of the medial malleolus articular surface; and tibial bimalleolar angle (TBM), an indicator of congenital or post-traumatic ankle morphology [4, 20, 21]. Global lower extremity alignment was assessed using the hip–knee–ankle angle (HKA) (Fig. 2). Ankle and subtalar joint alignment was evaluated using four parameters (Fig. 4): tibiotalar tilt angle (TTA), defined as the angle between the distal tibial articular surface and that of the talar dome; tibial plafond inclination angle (TPIA), defined as the angle between the distal tibial articular surface and a vertical line to the ground; talar inclination angle (TIA), defined as the angle between the talar dome and a vertical reference line [22]; and hindfoot alignment angle (HAA), defined as the angle between the tibial mechanical axis and a line from the lowest point of the calcaneus to the center of the talar dome [23]. The accuracy of HAA measurement on whole-leg anteroposterior radiographs may be affected by rotational artifacts and limited visualization of the calcaneus. Two orthopedic surgeons independently performed the radiographic measurements in two separate sessions 4 weeks apart. Each observer was blinded to the other’s measurements and patient data. Intra- and interobserver reliabilities were assessed using intraclass correlation coefficients, which demonstrated good or excellent agreement.

The normal range for each alignment parameter was defined as the 2.5th to 97.5th percentile values from the control group. The normal ranges were: −6.9° ≤ HKA ≤ 4.8°, −2.6° ≤ TTA ≤ 2.4°, 83° ≤ TPIA ≤ 96°, 83° ≤ TIA ≤ 96°, and −6.1° ≤ HAA ≤ 6.8°.

### Statistical analysis

The prevalence of ankle OA was determined, and baseline demographic and radiographic parameters were compared between patients with and without ankle OA. The Wilcoxon rank-sum test was used to compare continuous variables on the basis of their distribution (Shapiro–Wilk test) and homoscedasticity (*f*-test), whereas the chi-squared test was used to compare categorical parameters. Pre- and postoperative alignment parameters were compared using the Wilcoxon signed-rank test. Postoperative changes in the proportion of patients achieving normal alignment were also analyzed.

Multivariate logistic regression analysis was performed to identify independent factors associated with failure to achieve postoperative alignment normalization. Covariates included age, sex, BMI, varus knee alignment, and presence of ankle OA. Statistical significance was set at *p* < 0.05. Post hoc analysis confirmed adequate statistical power (0.94). All statistical analyses were performed with JMP version 17.2 (SAS Institute Inc., Cary, NC, USA).

## Results

According to the Takakura–Tanaka classification, 202 patients (61%) were classified as stage 0, 70 (21%) as stage 1, 49 (15%) as stage 2, 6 (2%) as stage 3a, 4 (1%) as stage 3b, and none as stage 4. Ankle OA, defined as stage ≥ 2, was observed in 59 of 331 patients (18%). Compared with patients without ankle OA, those with ankle OA had a higher proportion of female patients (86% versus 73%, *p* = 0.024), greater varus in TAS (87° versus 90°, *p* = 0.005), and greater TMM (24° versus 21°, *p* < 0.001) (Table 2).

**Table 2:** Comparison of patient demographics and morphological parameters between patients with and without ankle osteoarthritis (OA)

Parameters	Ankle OA (+) (*n* = 59 patients)	Ankle OA (−) (*n* = 272 patients)	*p*-Value^**^
Age^*^ (*years*)	73 (57–87)	76 (36–91)	0.149
Sex^†^ (male:female)	8 (14): 51 (86)	73 (27): 199 (73)	0.024
Body mass index^*^ (*kg/m*^*2*^)	26 (20–40)	25 (15–39)	0.170
Affected side^†^ (right:left)	26 (15): 33 (20)	142 (85): 130 (80)	0.257
Tibial anterior surface angle^*^ *(°)*	87 (79–103)	90 (79–104)	0.005
Tibial medial malleolus angle^*^ *(°)*	24 (13–53)	21 (4–44)	< 0.001
Tibial bimalleolus angle^*^ *(°)*	79 (66–103)	80 (68–101)	0.199

^*^Median (range); ^†^number (%); ^**^Wilcoxon rank-sum or chi-squared test

**Fig. 5:**
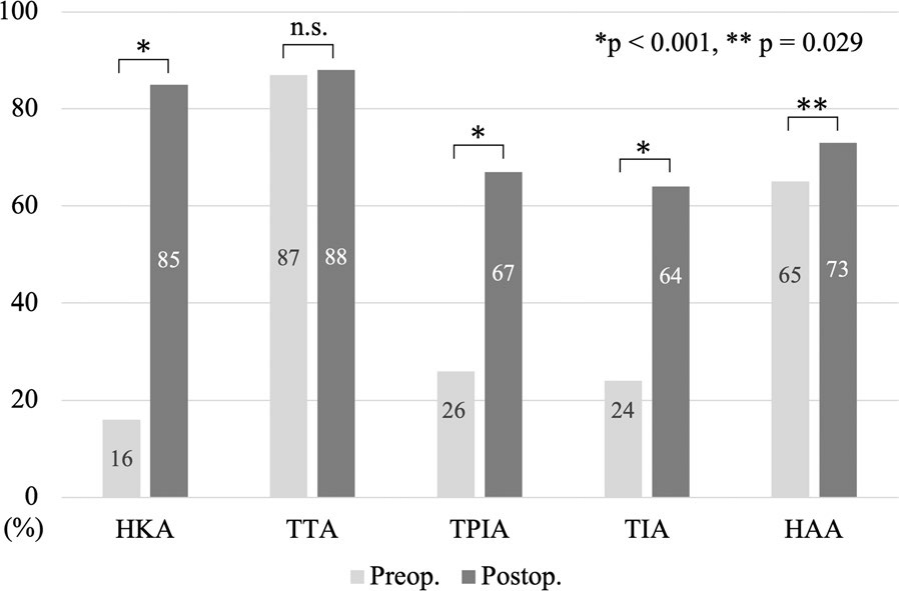
Postoperative changes in the proportion of patients falling within the normal range for each alignment parameter

The median values of the alignment parameters changed after TKA as follows: HKA improved from −12° to −2°, TTA from −0.2° to −0.1°, TPIA from 99° to 94°, TIA from 99° to 95°, and HAA from 0.2° to 0.7°. Significant improvements were observed in HKA, TPIA, and TIA (all *p* < 0.001), with TPIA and TIA shifting toward being parallel to the floor postoperatively (Table 3). The proportion of patients within the normal range increased postoperatively from 16% to 85% (*p* < 0.001) for HKA. Similarly, normalization rates increased significantly for TPIA (26% to 67%, *p* < 0.001), TIA (24% to 64%, *p* < 0.001), and HAA (65% to 73%, *p* = 0.029) (Fig. 5).

**Table 3:** Change in alignment parameters after total knee arthroplasty

Parameters^*^ (°)	Preoperative	Postoperative	*p*-Value^†^
Hip–knee–ankle angle	−12 (−32 to 32)	−2.0 (−20 to 11)	< 0.001
Tibiotalar tilt angle	−0.2 (−22 to 5)	−0.1 (−23 to 4)	0.424
Tibial plafond inclination angle	99 (66–116)	94 (79–112)	< 0.001
Talar inclination angle	99 (68–121)	95 (79–109)	< 0.001
Hindfoot alignment angle	0.2 (−33 to 21)	0.7 (−22 to 20)	0.140

^*^Median (range); ^†^Wilcoxon signed-rank test

Multivariate logistic regression analysis identified following three independent predictors of persistent postoperative malalignment: ankle OA for TTA (odds ratio [OR] = 6.62) (Table 4), female sex for TPIA (OR = 2.32) (Table 5) and TIA (OR = 3.19) (Table 6), and varus knee alignment for TIA (OR = 2.81) (Table 6). No factors were significantly associated with failure to normalize HAA (Table 7). In subgroup comparison, patients with ankle OA had a significantly lower rate of postoperative TTA normalization than those without OA (64% versus 93%, *p* < 0.001). Female patients were less likely to achieve normalization of TPIA (62% versus 79%, *p* = 0.001) and TIA (58% versus 81%, *p* < 0.001). In addition, patients with preoperative varus knee alignment had a lower rate of postoperative TIA normalization than those with valgus alignment (62% versus 80%, *p* = 0.007).

**Table 4:** Multivariate logistic regression analysis of predictors of abnormal tibiotalar tilt angle (TTA) after total knee arthroplasty (*R*^2^ = 0.12, *p* < 0.001)

Parameters	Estimate (standard error)	Odds ratio	*p*-Value
Age	−0.01 (2.32)	0.99	0.706
Sex (female)	0.11 (0.23)	1.25	0.620
Body mass index	0.02 (0.05)	1.02	0.603
Varus knee	0.02 (0.28)	1.05	0.933
Ankle osteoarthritis (+)	0.95 (0.18)	6.62	< 0.001

**Table 5:** Multivariate logistic regression analysis of predictors of abnormal tibial plafond inclination angle (TPIA) after total knee arthroplasty (*R*^2^ = 0.03, *p* = 0.047)

Parameters	Estimate (standard error)	Odds ratio	*p*-Value
Age	0.01 (0.02)	1.01	0.413
Sex (female)	0.42 (0.15)	2.32	0.006
Body mass index	0.00 (0.03)	1.00	0.959
Varus knee	0.20 (0.19)	1.48	0.294
Ankle osteoarthritis (+)	0.14 (0.15)	1.32	0.353

**Table 6:** Multivariate logistic regression analysis of predictors of abnormal talar inclination angle (TIA) after total knee arthroplasty (*R*^2^ = 0.06, *p* = 0.020)

Parameters	Estimate (standard error)	Odds ratio	*p*-Value
Age	0.00 (0.02)	1.00	0.869
Sex (female)	0.58 (0.16)	3.19	< 0.001
Body mass index	−0.01 (0.03)	0.99	0.647
Varus knee	0.52 (0.20)	2.81	0.007
Ankle osteoarthritis (+)	0.18 (0.15)	1.43	0.241

**Table 7:** Multivariate logistic regression analysis of predictors of abnormal hindfoot alignment angle (HAA) after total knee arthroplasty (*R*^2^ = 0.01, *p* = 0.536)

Parameters	Estimate (standard error)	Odds ratio	*p*-Value
Age	0.00 (0.01)	0.26	0.607
Sex (female)	−0.16 (0.15)	1.09	0.296
Body mass index	−0.03 (0.03)	1.06	0.302
Varus knee	−0.25 (0.18)	1.95	0.162
Ankle osteoarthritis (+)	−0.07 (0.32)	0.05	0.824

## Discussion

The main findings of this study were as follows: (1) the incidence of ankle OA in patients undergoing TKA was 18%, with a higher prevalence in females and characteristic morphological features including increased varus of the TAS and increased TMM; (2) improvement in HKA after TKA was associated with significant improvement in TPIA and TIA, both of which became more parallel to the ground; and (3) independent factors associated with failure to normalize hindfoot alignment included female sex, varus knee alignment, and pre-existing ankle OA.

To our knowledge, this is the first study to evaluate changes in hindfoot alignment after TKA using normal reference values. By establishing normative alignment parameters from a healthy control group, we were able to objectively assess whether the hindfoot shifted toward neutral after lower extremity realignment. This approach also allowed identification of patient-specific factors that hindered normalization. Coronal plane deformity of the hindfoot can alter ankle kinematics and contribute to the development and progression of OA [9]. From a biomechanical perspective, finite element analyses have shown that reductions in articular contact area increase joint stress [24] and that internal rotation, plantar flexion, and eversion of the ankle are associated with increased mechanical stress on the cartilage [25]. These findings suggest that restoring neutral alignment may provide a more favorable biomechanical environment for the ankle. Future studies integrating radiographic alignment with clinical data may help elucidate the pathophysiology of adjacent joint degeneration, including ankle OA.

Previous studies have estimated the prevalence of ankle OA in patients with knee OA to be 24–35% [4, 26]. Murray et al. reported that radiographic ankle OA was more common in females than in males [27]. In the present study, 18% of patients undergoing TKA had ankle OA, with a higher prevalence in females. This lower incidence compared with previous studies may reflect differences in race, lifestyle, or the predominance of primary versus secondary OA. For example, secondary OA is reported to account for more than 70% of ankle OA in the USA [28], whereas primary OA is more common in Japan. Our larger sample size (*n* = 331) compared with previous studies may also have influenced the prevalence estimates. Morphologically, patients with ankle OA in our cohort had varus TAS and increased medial malleolar inclination (TMM), which is consistent with previous studies that identified anterior tibial plafond inclination and varus deformity as characteristic features of ankle OA [20, 29, 30].

The subtalar joint plays a key role in compensating for varus and valgus knee deformities. Varus knee alignment often results in compensatory valgus alignment of the hindfoot and vice versa [13]. Jeong et al. observed that varus knee deformity is compensated by a combination of ankle varus and valgus alignment at the subtalar joint and talus–calcaneus complex, reporting a moderate correlation between coronal plane changes in the knee and subtalar joint [31]. Other studies have also shown that hindfoot alignment changes after correction of knee deformity with TKA [12, 31, 32]. In the present study, although HAA itself did not change significantly postoperatively, the proportion of patients within the normal HAA range increased significantly. This supports previous findings that HKA and HAA are not directly correlated because subtalar joint motion is more involved in compensatory mechanisms [31]. However, the measurement of HAA on whole-leg radiographs may be limited by calcaneal rotation and poor visibility of key landmarks, raising concerns regarding reliability, and its accuracy needs to be verified [13, 23, 31].

Several studies have suggested that progressive varus deformity of the knee alters the orientation of the ankle joint, increasing the valgus tilt of the talus and distal tibial plafond to the ground [33]. These changes may compromise ankle biomechanics. Xie et al. reported a correlation between knee malalignment and ankle degeneration, suggesting that tibial varus contributes to the progression of ankle OA [33]. In the present study, correction of HKA with TKA resulted in improved TPIA and TIA parallelism to the ground, and approximately 60% of patients achieved normalization of these parameters. These improvements may correlate with improved ankle function after TKA.

To our knowledge, no previous study has evaluated factors associated with failure to normalize hindfoot alignment after TKA. In this study, female sex, varus knee alignment, and ankle OA were independent predictors of persistent postoperative malalignment. These findings suggest that even with correction of HKA, normalization of ankle alignment may be limited in patients with these risk factors. Rühling et al. reported that persistent varus TTA and pre-existing ankle OA were associated with ankle pain after TKA [14]. Although we did not assess clinical outcomes, our findings may aid in preoperative counseling, surgical planning, and selection of alignment strategies (e.g., MA versus KA-TKA). For example, MA-TKA may be preferred in patients with abnormal ankle alignment and pain. Conversely, in patients with severe ankle OA, TKA may have a limited impact on ankle symptoms and alignment. While TKA generally yields favorable outcomes, rates of patient dissatisfaction remain higher than after total hip arthroplasty [34]. Identifying risk factors for suboptimal outcomes and tailoring surgical approaches accordingly may improve patient satisfaction.

As demonstrated in this study, TKA significantly affects the biomechanical alignment of the entire lower extremity, including the hindfoot. Correcting knee alignment may reduce the risk of adjacent joint degeneration and improve functional outcomes and quality of life. Preoperative assessment of lower extremity alignment using whole-leg standing radiographs is essential because it allows simultaneous evaluation of hip, knee, and ankle [35]. Our findings emphasize the importance of managing knee deformity within a holistic framework for lower extremity OA. Future studies should explore the relationship between alignment correction and ankle symptoms, and assess whether improvement in alignment translates into clinical benefits.

This study has several limitations. First, it is a retrospective study with a relatively short follow-up period, limiting our ability to assess long-term changes in alignment or clinical symptoms. Second, only coronal plane alignment was evaluated using two-dimensional radiographs. Because rotation and rotational and sagittal plane deformities also affect ankle mechanics, lateral views and three-dimensional imaging should be considered in future studies. Third, although we discussed the potential functional benefits of alignment normalization, clinical outcomes were not assessed in this study and further studies are warranted to validate this association. Lastly, this study focused solely on MA-TKA; the influence of alignment strategies such as kinematic alignment was not evaluated. Future longitudinal studies should assess the long-term impact of TKA on ankle alignment, OA progression, and clinical symptoms, particularly in patients with pre-existing ankle OA.

## Conclusions

This study demonstrated that MA-TKA leads to significant improvements in coronal ankle and subtalar alignment, particularly at the tibial plafond and talus. However, normalization of hindfoot alignment was not consistently achieved in all patients. Female sex, preoperative varus knee deformity, and pre-existing ankle OA were identified as independent risk factors for persistent postoperative malalignment. These findings underscore the biomechanical interdependence of the knee and hindfoot and highlight the importance of comprehensive lower extremity assessment in patients undergoing TKA. Surgeons should identify high-risk patients during preoperative planning and consider individualized alignment strategies, additional imaging, or targeted counseling to reduce the risk of residual hindfoot malalignment and optimize postoperative outcomes.

## Data Availability

The datasets generated during and/or analyzed during the current study are available from the corresponding author upon reasonable request.

## Abbreviations

TKA: Total knee arthroplasty
OA: Osteoarthritis
MA: Mechanical alignment
KA: Kinematically aligned
BMI: Body mass index
TAS: Tibial anterior surface angle
TMM: Tibial medial malleolus angle
TBM: Tibial bimalleolar angle
HKA: Hip–knee–ankle angle
TTA: Tibiotalar tilt angle
TPIA: Tibial plafond inclination angle
TIA: Talar inclination angle
HAA: Hindfoot alignment angle
OR: Odds ratio
